# Effects of supplemented culture media from solid-state fermented *Isaria cicadae* on performance, serum biochemical parameters, serum immune indexes, antioxidant capacity and meat quality of broiler chickens

**DOI:** 10.5713/ajas.19.0179

**Published:** 2019-08-03

**Authors:** Shaoshuai Liu, Wenjuan Yan, Chang Ma, Yajing Liu, Limin Gong, Crystal Levesque, Bing Dong

**Affiliations:** 1 State Key Laboratory of Animal Nutrition, College of Animal Science and Technology, China Agricultural University, Beijing 100193, China; 2 Zhejiang BioAsia Biomedical Co., Ltd., Pinghu, Zhejiang, 314200, China; 3 Monogastric Nutrition Department of Animal Science, College of Agriculture and Biological Sciences, South Dakota State University, Brookings, South Dakota 57007, USA

**Keywords:** Culture Media of Solid-state Fermented *Isaria cicadae*, Serum Biochemical Parameters, Serum Immune Indexes, Antioxidant Capacity, Meat Quality

## Abstract

**Objective:**

The objective of this study was to investigate effects of supplementation of culture media from solid-state fermented *Isaria cicadae* (*I. cicadae*) on performance, serum biochemical parameters, serum immune indexes, antioxidant capacity and meat quality of broiler chickens.

**Methods:**

A total of 648 Arbor Acres male broiler chickens(1 d; average body weight, 42.93± 0.47 g) were randomly assigned to 6 treatments, each with six replicates and 18 broiler chickens per replicate. Broiler chickens were fed phase I (d 1 to 21) and phase II (d 22 to 42) diets. The phase I diets were corn and soybean-meal based diets supplemented with 0%, 2%, 4%, 6%, 8%, or 10% culture media from solid-state fermented *I. cicadae* respectively. The phase II diets were corn and soybean-meal based diets supplemented with 0%, 1.33%, 2.67%, 4.00%, 5.32%, or 6.67% culture media from solid-state fermented *I. cicadae* respectively.

**Results:**

In phase I, the broiler chickens with the supplementation of culture media had increased body weight gain and feed intake (linear and quadratic, p<0.05) with increasing inclusion of culture media. The levels of serum total antioxidant capacity (T-AOC), glutathione (GSH) and superoxide dismutase (SOD) increased linearly (p<0.05). In phase II, levels of serum T-AOC and interleukin-1β increased linearly (p<0.05), and GSH increased (p<0.05). In the kidney, GSH and glutathione peroxidase (GSH-Px) concentrations increased (linear and quadratic, p<0.05) and SOD concentration increased linearly (p<0.05). Compared to the control, shear force and drip loss of breast muscle decreased (linear and quadratic, p< 0.05). Drip loss of leg muscle decreased linearly and quadratically (p<0.05).

**Conclusion:**

Dietary supplementation of culture media from solid-state fermented *I.cicadae* which was enriched in both wheat and residual bioactive components of *I. cicadae* enhanced the growth performance of broiler chickens. It also improved body anti-oxidative status and contributed to improve broiler meat quality.

## INTRODUCTION

*Isaria cicadae* (*I. cicadae*) is a famous and valuable traditional Chinese medicine. It is known as Chan Hua in China and is formed in cicada nymphs infected by *Paecilomyces cicadae* [1]. *I. cicadae* has many putative active functions such as: antitumor activity, analgesic activity and amelioration of renal function [2,3]. *I. cicadae* is an anamorph of *Cordyceps cicadae* which contains not only nutrients such as proteins, amino acids and various trace elements, but also various bioactive constituents such as sterols, cyclic peptides, nucleosides, and polysaccharides [4–6]. As market demand increases, natural supplies of *I. cicadae* have been widely depleted due to over harvesting, thus artificial culture of *I. cicadae* by inoculation of *I. cicadae* on plants (wheat or rice) provides a productive way to obtain large-scale *I. cicadae*. There are few studies reporting that feeding chickens with *I. cicadae* as a feed additive can improve immune functions [7] and muscle meat quality [8], while it is unlikely to be popularly applied in animal feed due to the high cost.

In the large scale production of artificial culture of *I. cicadae*, a large amount of discarded *I. cicadae* culture media are produced after harvesting the fruiting bodies of *I. cicadae* for medicinal usage. During the cultivation, mycelia and many bioactive ingredients are secreted into the culture media, which contains many nutrients and active ingredients [9]. Culture media of solid-state fermentation is usually composed of plant (wheat or rice) and residual components of *I. cicadae*. Measurement of the nutrient composition and active substances of a typical abandoned cultured medium reveals that it contains a considerate percentage of crude protein, crude fat, carbohydrate and gross energy (GE). The medium is also rich in bioactive components such as cordyceptin (adenosine), D-mannitol, N6-(2-hydroxyethyl), polysaccharides and numerous essential amino acids. Compared to the fruiting body of *I. cicadae*, the abandon media contains less protein, fat, fiber and bioactive components but more carbohydrates and GE. It demonstrates the abandon media of solid fermented *I. cicadae* is an excellent feed ingredient for animals.

In this study, we evaluate the effects of supplementation with *I. cicadae* waste media as a feed ingredient on the growth performance, serum biochemical parameters, serum immune parameters, antioxidant status and meat quality in broiler chickens.

## MATERIALS AND METHODS

### Chemical analysis of culture media

Culture media of *I. cicadae* was collected once the solid-state fermentation completed. The fermentation process was described briefly as following. The conidia of *I. cicadae* were collected from agar slants by sterilized distilled water washing. This liquid form of conidia was filtered followed by cultivation in seed culture media at a suitable concentration. Then 5 mL of the seed culture medium was inoculated to the medium of solid-state fermentation (mixture of 20 g wheat and 32 mL basal liquid supplement medium) and allowed to be incubated at 20°C for 12 d in the dark followed by the incubation at 23°C, 60% to 70% relative humidity and 500 lx 12 hrs/d for 12 to 20 d. The basal liquid contained sucrose, peptone, magnesium sulfate and monopotassium phosphate. The fruiting body of *I. cicadae* was harvested by cutting along the surface of wheat medium. The remaining wheat medium was the cultured medium of solid-state fermented *I. cicadae* used in this study.

Culture media of solid-state fermented *I. cicadae* was analyzed for crude protein, crude fat, calcium, and total phosphorus following AOAC 2013. The GE was determined by an Automatic Energy Analyzer (Parr 1281, Moline, IL, USA). AAs except for methionine and tryptophan were measured using ion-exchange chromatography with an Automatic Amino Acid Analyzer (L-8800; Hitachi Automatic Amino Acid Analyzer, Tokyo, Japan) after hydrolyzing with 6 mol/L HCl at 110°C for 24 h. Methionine was measured after oxidation with performic acid and subsequent hydrolysis with HCl while tryptophan was determined after alkaline hydrolysis at 120°C for 16 h (AOAC, 2013) with separation using reverse-phase High performance liquid chromatography (Waters 2690, Milford, MA, USA). Carbohydrates were measured by Sulfuric Acid–UV method [10]. soluble dietary fiber, insoluble dietary fiber and total dietary fiber were analyzed using the Ankom Dietary Fiber Analyzer (Ankom Technology, Macedon, NY, USA) according to a reported modified procedure [11]. Culture media from solid-state fermented *I. cicadae* was from Zhejiang BioAsia Biomedical Co., Ltd. (Pinghu, Zhejiang, China). Its nutritional contents are shown in Table 1.

### Animals and experiment design

A total of 648 Arbor Acres male broiler chickens (1 d; average body weight, 42.93±0.47 g) were purchased from Arbor Acres Poultry Breeding Company (Beijing, China). On d 1, all birds were randomly assigned to 6 treatments, each with six replicates and 18 broiler chickens per replicate. Broiler chickens were fed phase I (d 1 to 21) and phase II (d 22 to 42) diets. The experiment used a corn and soybean-meal based diet which was supplemented with 0%, 2%, 4%, 6%, 8%, or 10% culture medium of solid-state fermented *I. cicadae* in phase I and 0, 1.33%, 2.67%, 4.00%, 5.32%, or 6.67% in phase II for groups 1 to 6, respectively. All diets were fed in mash form. All nutrients contained in the basal diet met or exceeded the requirements suggested by the Chinese broiler breeding standard (Standard number NY/T33-2004). The composition of the diets is shown in Table 2.

The experiment was conducted at National Feed Engineer ing Technology Research Centre (Chengde, Hebei, China). All chicks were kept under the same hygienic, managerial and environmental conditions. The temperature in the house was initially at 34°C after which the temperature was gradually reduced by 2°C a week until it reached 24°C and then maintained at this temperature until the end of the 42-d experiment. All broiler chickens had *ad libitum* access to feed and water. All chicks were inoculated with inactivated Newcastle Disease Vaccine on d 7 and d 21 and inactivated Infectious Bursal Disease Vaccine on d 14 and d 28.

### Collection of performance data

Feed consumption and body weight of broiler chickens for each replicate after fasting for 12 h were measured on d 21 and d 42. Body weight gain (BWG), feed intake (FI), and feed conversion ratio (FCR, F:R) were calculated during 1 to 21 d, 22 to 42 d, and 1 to 42 d.

### Slaughter and sample collection

On d 21 and d 42, three broiler chickens whose body weights were close to average body weight were randomly selected from each replicate pen in each treatment and their blood sampled. Serum was collected by centrifugation at 1,500×*g* for 10 min and stored at −20°C for later analysis.

After blood sampling on d 42, one broiler per pen was bled from the jugular vein and decapitated. After slaughter, liver and kidney were sampled, placed in liquid nitrogen and stored at −80°C for the analysis of antioxidant capacity. Muscles of left breast and leg were sampled for analysis of meat quality.

### Assay of serum biochemical parameters

The concentrations of serum alanine aminotransferase (ALT), aspartate aminotransferase (AST), total protein (TP), albumin (ALB), glucose (GLU), total bilirubin (TBiL), alkaline phosphatase (ALP), and uric acid (UA) were measured by corresponding commercial kits (BioSino Bio-technology and Science Inc., Beijing, China) using automatic biochemical analyzer (Hitachi 7160, Hitachi High-Technologies Corporation, Japan).

### Assay of serum immune indexes

The concentrations of serum immunoglobulin A (IgA), IgG, IgM, interleukin-1β (IL-1β) and interferon-γ (IFN-γ) were measured with corresponding commercial kits (Beijing Sino-UK Institute of Biological Technology, Beijing, China) using automatic biochemical analyzer (Hitachi 7160, Hitachi High-Technologies Corporation, Japan).

### Antioxidant capacity

Serum, liver and kidney were analyzed for total antioxidant capacity (T-AOC), glutathione (GSH), glutathione peroxidase (GSH-Px), superoxide dismutase (SOD) and malondialdehyde (MDA) by commercial kits (Jiancheng Biochemical Reagent Co., Nanjing, China) according to manufacturer’s instructions.

### Meat quality

At 45 min postmortem, the pH_45min_ of the breast and leg muscles was detected using an SPK pH meter (pH-star, DK2730, Herlev, Denmark). Meanwhile, the pH_24h_ was measured at 24 h postmortem in a chilling room.

Meat color was measured at 45 min post-mortem using a Minolta chromameter (CR-410, Konica minota, Tokyo, Japan). The values were described as L* (lightness), a* (redness), and b* (yellowness). Drip loss was measured as described previously [12,13].

After 24 h post-mortem, samples were used to measure shear force value [14]. Briefly, samples were stored in a vacuum bag, and heated in a water bath at 75°C until the internal temperature of the sample reached 70°C. After cooling under running water, the surface water of samples was removed. Then 10 cylindrical muscle core samples were collected (10 mm diameter×10 mm length) from each meat sample, and then were cut parallel to the fiber orientation using a cylindrical cutter. Peak shear force was determined by using a digital-display-muscle tenderness meter (C-LM3B, Tenovo, Harbin, China).

### Statistical analysis

Data were analyzed using one-way analysis of variance in accordance with the general linear model procedures of SAS 9.4 (SAS Institute Inc., Cary, NC, USA) as a completely randomized design including supplementation levels as the fixed effect. Statistical differences among mean values were assessed using Duncan’s multiple range test. The pen was deemed as one experimental unit. The model included dietary treatment as the only fixed effect. Interactive matrix algebra procedure of SAS was adopted to generate the coefficients of unequally spaced contrasts. Subsequently, the linear and quadratic responses to levels of culture medium from solid-state fermented *I. cicadae* were assessed by the orthogonal polynomial contrast. Significance level was set at p<0.05.

## RESULTS

### Production performance

The effects of culture media from solid-state fermented *I. cicadae* on the performance of broiler chickens are shown in Table 3. The broiler chickens fed with culture media of *I. cicadae* demonstrated increased BWG and FI as the supplementation increased in phase I (linear and quadratic, p< 0.05). Supplementing culture medium showed no significant effect on FCR and mortality rate in phase I, nor FI, BWG, FCR and mortality in phase II and the entire phase (d 1 to 42).

### Serum biochemical parameters

No significant difference was observed in the levels of serum ALT, AST, TP, ALB, GLU, TBiL, ALP, and UA in phase I by dietary supplementation of culture media of *I. cicadae* (Table 4). In phase II, serum AST levels increased quadratically (p< 0.05). Meanwhile, serum ALP increased linearly (p<0.05) as the level of culture media increased.

### Serum immune indexes

Supplementation of culture medium from solid-state fermented *I. cicadae* increased serum IL-1β levels in both phase I and phase II (p<0.05, linearly p<0.05). Serum IgA in phase I increased (p<0.05) and tended to increase in phase II (p = 0.09). Serum IgG tended to increase as the supplementation increased in phase I (p = 0.08). Levels of serum IgM and IFN-γ were not different in both two phases (Table 5).

### Antioxidant capacity

In phase I, serum T-AOC, GSH, and SOD concentrations all increased linearly (p<0.05) with the increased supplemented level of culture media of *I. cicadae*, but no differences were observed in serum GSH-Px and MDA concentrations. In phase II, serum T-AOC concentration increased linearly (p<0.05) with the increased supplemented level of culture media of *I. cicadae*, and serum GSH concentration significantly increased (p<0.05), but no significant effects on serum GSH-Px, SOD, and MDA levels were observed (Table 6).

In the kidney, GSH and GSH-Px concentrations increased (linear and quadratic, p<0.05) with the increased inclusion of culture media. Kidney SOD concentrations increased linearly (p<0.05). There were no differences in T-AOC and MDA concentrations among treatments. In the liver, concentrations of GSH-Px and MDA showed slightly differences (p = 0.05) among treatments and showed a quadratic response (p<0.05 and p = 0.07) as the supplementation of culture media increased. There were no differences in T-AOC, GSH, and total SOD concentrations among groups (Table 7).

### Meat quality

a* and pH_45min_ values of the breast muscle tended to decrease quadratically (p<0.05) (Table 8). The shear force and drip loss decreased (linear and quadratic, p<0.05) as the culture medium supplementation increased. There was no significant difference in the values of L*, b*, and pH24h. In the leg muscle, drip loss decreased (linear and quadratic, p<0.05), but supplementing diets with different levels of culture media of solid-state fermented *I. cicadae* did not alter the values of L*, a*, b*, pH_45min_, pH_24h_, and shear force significantly.

## DISCUSSION

Intensive selection for husbandry animals has concentrated on growth rather than meat quality. In China, city people prefer to consume village raised native broiler chickens. These broiler chickens take longer to finish before going to market thus have more flavor in their meat. Tibet village broiler chickens uncaged raised in Tibetic plateau are increasingly commercially popular because these broiler chickens are Cordyceps-eating and better tasting. Few studies feeding cordyceps in diets to chickens have been conducted, which demonstrate improved growth performance and deposit of meat nutrients. But feeding chickens with cordyceps is not realistic because of its high cost.

Culture media from solid-state fermented *I. cicadae* contains both plant stoma, usually wheat or rice, and residual bioactive components of *I. cicadae*. This makes the culture media potentially a good candidate as an ingredient for broiler feed. In this study, supplementation of culture media from solid-state fermented *I. cicadae* to broiler chickens, compared to the un-supplemented group, resulted in an increased BWG in phase I (d 1 to 21), improved antioxidant status and meat quality with increasing inclusion of the media.

Many plants and fungi can simultaneously improve the body antioxidant status and enhance immune responses. This is probably due to the natural polysaccharides in these herbs [15]. Polysaccharides in Cordyceps are water soluble (CPS-2) [16]. Its predicted structure consists of a repeating unit of α-(1,4)-D-glucose and α-(1,3)-D-mannose, branched with α-(1,4,6)-D-glucose [16]. A Cordyceps polysaccharide is proved to protect neurons from free radicals [17]. In this study, supplementation of culture medium from fermented *I. cicadae* improved antioxidant status marked by increased serum and tissue T-AOC, GSH, GSH-Px levels. This contributed improved meat quality of breast and leg muscles which was reflected by the parameters of meat postmortem pH, water holding capacity, drip loss, meat color, and shear force. The culture media from fermented *I. cicadae* containing residual bioactive components of *I. cicadae*, significantly improved meat shear force (breast muscle) and drip loss (breast muscle and leg muscle). Oxidation status is closely related to meat water holding capacity because the cell membrane is composed of unsaturated fatty acids. Oxidation of these fatty acids, in particular, disrupts the membrane structure and fluidity. When the membrane phospholipids are oxidized, cell permeability is altered leading to decreased muscle water-holding capacity. Low drip loss means high meat water holding capacity and high water holding capacity can bring advantages in meat processing and meat appearance for consumers [18]. To protect against oxidative injuries, body anti-oxidative system consisting of anti-oxidative enzymes: SOD, GSH-Px, and catalase which catalyze dismutation of superoxide anions to hydrogen peroxide, the degradation of H_2_O_2_ and hydroperoxides originating from unsaturated fatty acids at the expense of reduced glutathione, and convert H_2_O_2_ into water [19]. There are numerous components showing anti-oxidation activity in *I. cicadae*, like cordycepic acid (D-mannitol) [20], cordycepin (3′-deoxyadenosine) [21–23] and cordyceps polysaccharide [24,25]. Others reported antioxidant compounds like isoflavone [26], selenium [27], arginine [28], resveratrol [29], and vitamin E [30] can also be found in *I. cidadae*. These components all contributed to improve the quality of the broiler meat as antioxidants. This is important for producing competitive meat product for market.

Meat color is reflected by the lightness, redness and yellow ness (L*, a*, and b*). Previous reports revealed that muscle fiber type was closely related to meat color. The muscles enriched in mitochondria are slow-oxidative type I red muscles like leg muscle which contains more myoglobin, while the muscles active in glycolytic types are white muscles like breast muscle. Physical training [31,32] and some nutrient factors [29] are reported to change muscle type. In this study, we did observe higher redness value of breast muscle which is affected by muscle myoglobin contents [33] of broiler chickens by feeding cultured medium of fermented *I. cicadae* to broiler chickens.

Fungi polysaccharides have activities beyond being as antioxidants. A selenium polysaccharide from Cordyceps mycelium showed significant antioxidant activity *in vivo* in mice, thus it suggested uses as a potential antioxidant enhancing adaptive immune response [34]. Polysaccharide purified from fermentation medium of cordyceps showed stimulation effects on macrophages [35,36] and mononuclear cells [37]. Other bioactive components, cordycepin, cordycepic acid, myriocin and beauvericin in *I. cicadae*, are reported to inhibit the growth of carcinoma cells [38]. Cordycepin resists the growth of hepatocarcinoma via PI3K/Akt/mTOR and Nrf2/HO-1/NF-κB pathway [39]. In this study, feeding broiler chickens with culture media from solid-state fermented *I. cicadae* in diets increased serum IL-1β levels during phase I (d 1 to 21) and phase II (d 22 to 42), suggesting it promoted cellular immune responses. The enhanced immune system likely enabled the spared energy from immune defense to be used for growth thus increased BWG of broiler chickens. IL-1β is a member of interleukin family of cytokines. It can trigger complex reactions of combinatorial phosphorylation and ubiquitination activating nuclear factor kb signaling and JNK to induce the expression of IL-1 target genes (IL-6, IL-8, NF-κB inhibitory protein [Iκ-B]) [40]. Macrophages and monocytes, as the sentinel cells of the innate immune system, are the major source of IL-1β. Other immune relevant cells such as epithelial cells, endothelial cells and fibroblasts can also produce IL-1β [41–43]. The main function of IL-1β is to control proinflammatory reactions in response to tissue injury by bacteria or virus [44–46].

## CONCLUSION

Taken together, dietary supplementation of culture media from solid-state fermented *I. cicadae* which was enriched in both plant and residual bioactive components of *I. cicadae* enhanced the growth performance of broiler chickens. It also improved body anti-oxidative status and contributed to improve shear force and drip loss of broiler leg and breast muscles. Culture media from solid-state fermented *I. cicadae* could be a potential ingredient for broiler feed.

## Figures and Tables

**Table 1:** Nutritional and bioactive components of solid-state fermented *Isaria cicadae* (cultured medium and fruiting body, respectively)

Items	Solid-state cultured medium	Fruiting body
Nutritional components		
Gross energy (MJ/kg)	15.48	13.8
Protein (g/100 g)	15.61	30.1
Crude fat (g/100 g)	1.4	5.2
Saturated fatty acids (g/100 g)	0.3	1.31
Unsaturated fatty acids (g/100 g)	1.4	2.11
Carbohydrates (g/100 g)	70.7	26.7
Total fiber (g/100 g)	6.8	27.74
Soluble fiber (g/100 g)	4.4	2.49
Calcium (g/100 g)	0.11	2.1
Total phosphorus (g/100 g)	0.32	1.1
Bioactive components		
D-mannitol (mg/g)	26.56	60
Adenosine (mg/100 g)	10.8	110
N6-(2-hydroxyethyl) (mg/100 g)	43	130
Polysaccharides (mg/g)	210.44	45
Amino acid contents (g/100 g)		
Methionine	0.17	0.22
Cystine	0.23	0.44
Lysine	0.31	0.94
Threonine	0.44	1.09
Tryptophan	0.13	1.53
Arginine	0.52	1.12
Isoleucine	0.36	0.44
Leucine	0.62	1.49
Valine	0.53	0.84
Histidine	0.19	0.39
Phenylalanine	0.43	0.74
Glycine	0.46	1.01
Serine	0.51	1.16
Proline	1.02	1.93
Alanine	0.485	1.54
Aspartic acid	0.76	2.05
Glutamate	2.14	2.31

**Table 2:** Ingredient composition and nutrient levels of experimental diets (as-fed basis, %)

Items	Groups1)					

	1	2	3	4	5	6
1 to 21 d						
Ingredients						
Corn	59.96	56.83	55.92	53.48	54.49	53.20
Soybean meal	30.39	32.10	30.58	31.07	26.72	25.46
Fish meal	3.68	2.41	3.10	2.59	5.00	5.55
Cultured medium of *I. cicadae*	0.00	2.00	4.00	6.00	8.00	10.00
Soybean oil	2.28	2.75	2.59	2.98	2.30	2.31
Dicalcium phosphate	1.44	1.59	1.53	1.58	1.32	1.32
Limestone	1.18	1.24	1.20	1.22	1.10	1.09
Sodium chloride	0.38	0.38	0.38	0.38	0.38	0.38
DL-methionime (98.5%)	0.19	0.20	0.20	0.20	0.19	0.19
Premix2)	0.50	0.50	0.50	0.50	0.50	0.50
Total	100.00	100.00	100.00	100.00	100.00	100.00
Nutrient levels3)						
ME (Mcal/kg)	3.00	3.00	3.00	3.00	3.00	3.00
Crude protein (%)	21.53	21.56	21.53	21.58	21.57	21.59
Calcium (%)	1.02	0.98	1.15	1.01	1.12	1.01
Total phosphorus (%)	0.69	0.69	0.72	0.70	0.70	0.69
Lysine (%)	1.26	1.25	1.29	1.26	1.21	1.25
Methionine (%)	0.57	0.56	0.56	0.56	0.57	0.57
22 to 42 d						
Ingredients						
Corn	59.95	58.71	57.51	56.29	55.20	54.05
Soybean meal	31.83	31.55	31.32	31.07	30.82	30.58
Fish meal	0.40	0.44	0.44	0.45	0.44	0.43
Cultured medium of *I.cicadae*	0.00	1.33	2.67	4.00	5.32	6.67
Soybean oil	4.16	4.30	4.40	4.53	4.55	4.61
Dicalcium phosphate	1.40	1.43	1.43	1.43	1.43	1.43
Limestone	1.23	1.21	1.20	1.20	1.20	1.20
Sodium chloride	0.38	0.38	0.38	0.38	0.38	0.38
DL-methionime (98.5%)	0.15	0.15	0.15	0.15	0.15	0.15
Premix2)	0.50	0.50	0.50	0.50	0.50	0.50
Total	100.00	100.00	100.00	100.00	100.00	100.00
Nutrient levels3)						
ME (Mcal/kg)	3.10	3.10	3.10	3.10	3.10	3.10
Crude protein (%)	20.28	20.13	20.12	20.15	20.08	20.08
Calcium (%)	0.97	0.89	0.94	0.96	0.91	0.93
Total phosphorus (%)	0.66	0.65	0.67	0.66	0.68	0.66
Lysine (%)	1.19	1.21	1.07	1.12	1.21	1.15
Methionine (%)	0.48	0.48	0.48	0.46	0.47	0.45

ME, apparent metabolizable energy.

1) The experiment applied a corn and soybean-meal based diet which was supplemented with 0%, 2%, 4%, 6%, 8%, or 10% culture media of solid-state fermented *Isaria cicadae* in phase I (from d 1 to 21) for groups 1 to 6 respectively, and 0%, 1.33%, 2.67%, 4.00%, 5.32%, or 6.67% in phase II (from d 22 to 42) for groups 1 to 6.

2) Premix provided the following per kg of complete diet: vitamin A, 10,000 IU; vitamin D_3_, 2,750 IU; vitamin E, 30 IU; vitamin K_3_, 2 mg; vitamin B_1_, 1.5 mg; vitamin B_2_, 6 mg; vitamin B_6_, 3 mg; vitamin B_12_, 12 μg; nicotinic acid, 40 mg; pantothenic acid, 12 mg; folic acid, 0.8 mg; biotin, 0.2 mg; Zn, 100 mg; Mn, 150 mg; Fe, 95 mg; Cu, 10 mg; I, 1.25 mg; Se, 0.3 mg.

3) All nutrient levels except metabolizable energy were analysed.

**Table 3:** Effects of graded levels of culture media of solid-state fermented *Isaria cicadae* on growth performance in broiler chickens

Items	Groups1)						SEM	p value		
	
	1	2	3	4	5	6		ANOVA	Linear	Quadratic
Body weight, initial	42.9	42.9	43.1	42.9	42.9	43.0	0.16	0.97	0.94	0.92
21 d (g)	822.9^c^	839.1^bc^	840.7^bc^	863.4^a^	854.9^ab^	836.3^bc^	4.45	<0.01	<0.01	<0.01
42 d (g)	2,307	2,356.6	2,349.3	2,345.9	2,367.2	2,308.5	17.06	0.08	0.95	<0.01
1 to 21 d										
Feed intake (g/bird)	1,044^b^	1,069^ab^	1,076^ab^	1,094^a^	1,083^ab^	1,066^ab^	9.4	0.02	0.04	<0.01
Body weight gain (g/bird)	780^c^	796^bc^	798b^c^	820^a^	812^ab^	793^bc^	4.4	<0.01	0.04	<0.01
F:G	1.34	1.34	1.35	1.33	1.33	1.34	0.01	0.92	<0.01	<0.01
Mortality rate (%)	2.78	2.78	1.85	0.93	0.93	0.93	1.49	0.86	0.23	0.79
22 to 42 d										
Feed intake (g/bird)	2,932	3,000	3,026	3,020	3,056	2,927	64.3	0.67	0.90	0.07
Body weight gain (g/bird)	1,486	1,517	1,509	1,483	1,512	1,472	15.3	0.23	0.70	0.02
F:G	1.98	1.98	2.00	2.05	2.03	1.99	0.05	0.86	0.65	0.56
Mortality rate (%)	0.00	0.00	0.00	0.00	0.00	0.00	-	-	-	-
1 to 42 d										
Feed intake (g/bird)	3,973	4,070	4,101	4,100	4,139	3,997	61.7	0.39	0.53	0.04
Body weight gain (g/bird)	2,265	2,314	2,306	2,303	2,324	2,265	16.8	0.08	0.85	0.02
F:G	1.76	1.76	1.78	1.78	1.78	1.77	0.03	0.98	0.64	0.50
Mortality rate (%)	2.78	2.78	1.85	0.93	0.93	0.93	1.49	0.86	0.23	0.79

Values are the means of six replicate pens of 18 birds in each treatment (n = 108).

SEM, standard error of the mean; ANOVA, analysis of variance; F:G, the ratio of feed to gain.

1) The experiment applied a corn and soybean-meal based diet which was supplemented with 0%, 2%, 4%, 6%, 8%, or 10% culture media of solid-state fermented *Isaria cicadae* in phase I (from d 1 to 21) for groups 1 to 6 respectively, and 0%, 1.33%, 2.67%, 4.00%, 5.32% or 6.67% in phase II (from d 22 to 42) for groups 1 to 6.

a–c In the same row, values with different letter superscripts mean significant difference (p<0.05), while with the same or no letter superscripts mean no significant difference (p>0.05).

**Table 4:** Effects of graded levels of culture media of solid-state fermented *Isaria cicadae* on serum biochemical parameters in broiler chickens

Items	Groups1)						SEM	p value		
	
	1	2	3	4	5	6		ANOVA	Linear	Quadratic
21 d										
ALT (U/L)	10.6	8.15	6.28	6.73	10.87	11.67	2.17	0.37	0.45	0.05
AST (U/L)	138.6	151.4	140.0	148.4	177.4	147.3	18.0	0.69	0.36	0.73
TP (g/L)	22.82	23.22	24.03	20.55	22.53	25.32	2.31	0.79	0.70	0.41
ALB (g/L)	9.12	9.33	9.43	8.02	9.23	9.57	0.87	0.83	0.94	0.50
GLU (mmol/L)	11.21	11.64	10.96	9.83	10.69	10.68	0.92	0.82	0.33	0.59
TBiL (μmol/L)	8.77	10.27	9.45	8.72	8.35	9.82	0.83	0.56	0.85	0.82
ALP (μmol/L)	4,741	48,522	5,415	4,883	5,053	4,874	270.8	0.57	0.76	0.25
UA (μmol/L)	254.7	229.2	238.2	173.0	194.0	234.2	31.41	0.46	0.33	0.22
42 d										
ALT (U/L)	4.77	5.24	5.20	6.08	6.42	7.03	0.70	0.22	0.02	0.74
AST (U/L)	191.03^b^	254.87^ab^	292.62^a^	255.80^ab^	230.27^ab^	208.65^ab^	21.05	0.03	0.90	<0.01
TP (g/L)	30.93	30.53	28.2	24.58	27.83	28.37	2.07	0.33	0.17	0.17
ALB (g/L)	11.47	12.33	11.22	9.7	11.68	10.82	0.57	0.053	0.17	0.48
GLU (mmol/L)	11.33	13.3	10.84	10.42	12.00	11.04	0.85	0.23	0.45	0.85
TBiL (μmol/L)	9.97	10.05	9.57	9.45	10.33	10.2	0.62	0.9	0.67	0.38
ALP (μmol/L)	1,930^b^	1,924^b^	2,039^b^	3,512^a^	2,185^b^	3,042^a^	156.8	<0.01	<0.01	0.32
UA (μmol/L)	220.33	255.67	226.33	215.67	227.00	156.33	31.33	0.38	0.09	0.17

Values are the means of one broiler selected from each replicate pen in one treatment (n = 6).

SEM, standard error of the mean; ANOVA, analysis of variance; ALT, alaine aminotransferase; AST, aspartate aminotransferase; TP, total protein; ALB, albumin; GLU, glucose; TBiL, total bilirubin; ALP, alkaline phosphatase; UA, uric acid.

1) The experiment applied a corn and soybean-meal based diet which was supplemented with 0%, 2%, 4%, 6%, 8%, or 10% culture media of solid-state fermented *Isaria cicadae* in phase I (from d 1 to 21) for groups 1 to 6 respectively, and 0%, 1.33%, 2.67%, 4.00%, 5.32%, or 6.67% in phase II (from d 22 to 42) for groups 1 to 6.

a,b In the same row, values with different letter superscripts mean significant difference (p<0.05), while with the same or no letter superscripts mean no significant difference (p>0.05).

**Table 5:** Effects of graded levels of culture media of solid-state fermented *Isaria cicadae* on serum immune indexes in broiler chickens

Items	Groups1)						SEM	p value		
	
	1	2	3	4	5	6		ANOVA	Linear	Quadratic
21 d										
IgA (g/L)	0.83^b^	0.92^ab^	1.18^a^	1.10^a^	0.96^ab^	1.06^a^	0.05	0.04	0.13	0.49
IgG (g/L)	5.63	5.68	6.36	6.09	5.97	6.07	0.12	0.08	0.26	0.51
IgM (g/L)	0.74	0.75	0.93	0.82	0.71	0.73	0.05	0.31	0.36	0.16
IL-1β (pg/mL)	47.54^b^	50.53^ab^	51.61^ab^	54.14^a^	54.77^a^	57.50^a^	1.01	0.03	<0.01	0.46
IFN-γ (pg/mL)	17.23	17.98	19.56	19.85	18.39	19.01	0.28	0.09	0.36	0.05
42 d										
IgA (g/L)	1.16	1.16	1.22	1.10	1.18	1.15	0.05	0.09	0.22	0.18
IgG (g/L)	7.31	7.21	7.35	7.48	7.18	7.46	1.50	0.26	0.18	0.24
IgM (g/L)	0.89	0.86	0.81	0.93	1.21	0.99	0.05	0.20	0.19	0.25
IL-1β (pg/mL)	58.13^b^	59.45^b^	60.13^b^	61.96^ab^	72.51^a^	72.49^a^	2.25	<0.01	<0.01	0.35
IFN-γ (pg/mL)	17.55	17.61	17.32	18.63	18.21	17.89	0.52	0.35	0.48	0.04

Values are the means of three broiler chickens from each replicate pen in one treatment (n = 18).

SEM, standard error of the mean; ANOVA, analysis of variance; Ig, immunoglobulin; IL-1β, interleukin-1 β; IFN-γ, interferon-γ.

1) The experiment applied a corn and soybean-meal based diet which was supplemented with 0%, 2%, 4%, 6%, 8%, or 10% culture media of solid-state fermented *Isaria cicadae* in phase I (from d 1 to 21) for groups 1 to 6 respectively, and 0%, 1.33%, 2.67%, 4.00%, 5.32%, or 6.67% in phase II (from d 22 to 42) for groups 1 to 6.

a,b In the same row, values with different letter superscripts mean significant difference (p<0.05), while with the same or no letter superscripts mean no significant difference (p>0.05).

**Table 6:** Effects of graded levels of culture media of solid-state fermented *Isaria cicadae* on serum antioxidant capacity in broiler chickens

Items	Groups1)						SEM	p value		
	
	1	2	3	4	5	6		ANOVA	Linear	Quadratic
21 d										
T-AOC (U/mL)	3.74^d^	4.56^cd^	5.17^bc^	5.28^bc^	6.80^ab^	7.91^a^	0.36	<0.01	<0.01	0.34
GSH (μmol/L)	9.31^c^	9.12^c^	10.51^bc^	11.41^ab^	13.85^a^	12.98^a^	0.58	<0.01	<0.01	0.82
GSH-Px (U/mL)	776.14	722.75	715.67	733.51	702.82	701.23	26.54	0.12	0.02	0.46
T-SOD (U/mg)	320.01^b^	319.32^b^	370.22^a^	379.90^a^	398.61^a^	390.76^a^	13.48	<0.01	<0.01	0.18
MDA (nmol/mL)	3.85	3.59	3.42	3.97	4.12	3.65	0.39	0.24	0.36	0.17
42 d										
T-AOC (U/mL)	3.88b^c^	4.34^ab^	3.59^c^	5.45^a^	5.31^a^	5.74^a^	0.35	<0.01	<0.01	0.59
GSH (μmol/L)	6.01^b^	8.23^a^	9.92^a^	8.54^a^	8.87^a^	10.82^a^	0.53	<0.01	0.84	0.39
GSH-Px (U/mL)	700.32	747.18	728.54	734.87	732.54	786.60	31.03	0.89	0.63	0.87
T-SOD (U/mg)	346.65	373.23	364.18	371.20	370.19	369.51	17.52	0.83	0.69	0.58
MDA (nmol/mL)	3.52	3.75	3.38	3.50	3.19	3.82	0.62	0.99	0.68	0.71

Values are the means of three broiler chickens from each replicate pen in one treatment (n = 18).

SEM, standard error of the mean; ANOVA, analysis of variance; T-AOC, total antioxidant capacity; GSH, glutathione; GSH-Px, glutathione peroxidase; T-SOD, total superoxide dismutase; MDA, malondialdehyde.

1) The experiment applied a corn and soybean-meal based diet which was supplemented with 0%, 2%, 4%, 6%, 8%, or 10% culture media of solid-state fermented *Isaria cicadae* in phase I (from d 1 to 21) for groups 1 to 6 respectively, and 0%, 1.33%, 2.67%, 4.00%, 5.32%, or 6.67% in phase II (from d 22 to 42) for groups 1 to 6.

a–d In the same row, values with different letter superscripts mean significant difference (p<0.05), while with the same or no letter superscripts mean no significant difference (p>0.05).

**Table 7:** Effects of graded levels of culture media of solid-state fermented *Isaria cicadae* on tissues antioxidant capacity in broiler chickens

Items	Groups1)						SEM	p value		
	
	1	2	3	4	5	6		ANOVA	Linear	Quadratic
Kidney										
T-AOC (U/mg)	2.06	2.09	2.03	1.83	2.26	2.14	0.19	0.73	0.67	0.50
GSH (μmol/g)	5.44^c^	5.43^c^	6.20^c^	6.13^c^	8.49^b^	10.67^a^	0.49	<0.01	<0.01	<0.01
GSH-Px (U/mg)	547.66^c^	696.71^bc^	816.95^ab^	834.78^ab^	875.62^a^	885.21^a^	41.54	<0.01	<0.01	0.01
T-SOD, (U/mg)	317.18^b^	324.23^b^	369.67^a^	374.30^a^	389.61^a^	384.86^a^	<0.01	0.01	<0.01	0.19
MDA (nmol/mL)	2.16	1.95	1.76	1.58	1.46	2.04	0.21	0.16	0.04	1.30
Liver										
T-AOC (U/mg)	1.69	1.76	1.80	1.81	1.69	1.78	0.08	0.83	0.75	0.51
GSH (μmol/g)	5.63	5.86	4.73	4.94	4.97	4.29	0.55	0.36	0.05	0.99
GSH-Px (U/mg)	847.94^ab^	827.28^ab^	773.45^b^	901.51a	901.17^a^	876.94^a^	30.69	0.05	0.33	0.03
T-SOD (U/mg)	362.61	375.94	348.78	365.95	367.10	377.46	17.67	0.88	0.66	0.54
MDA (nmol/mL)	2.16^ab^	2.17^ab^	2.79^a^	2.37^ab^	2.08^b^	2.28^ab^	0.16	0.05	0.94	0.07

Values are the means of one broiler selected from each replicate pen in one treatment (n = 6).

SEM, standard error of the mean; ANOVA, analysis of variance; T-AOC, total antioxidant capacity; GSH, glutathione; GSH-Px, glutathione peroxidase; T-SOD, total superoxide dismutase; MDA, malondialdehyde.

1) The experiment applied a corn and soybean-meal based diet which was supplemented with 0%, 2%, 4%, 6%, 8%, or 10% culture media of solid-state fermented *Isaria cicadae* in phase I (from d 1 to 21) for groups 1 to 6 respectively, and 0%, 1.33%, 2.67%, 4.00%, 5.32%, or 6.67% in phase II (from d 22 to 42) for groups 1 to 6.

a–c In the same row, values with different letter superscripts mean significant difference (p<0.05), while with the same or no letter superscripts mean no significant difference (p>0.05).

**Table 8:** Effects of graded levels of culture media of solid-state fermented *Isaria cicadae* on meat quality in broiler chickens

Items1)	Groups2)						SEM	p value		
	
	1	2	3	4	5	6		ANOVA	Linear	Quadratic
Breast muscle										
L*	49.73	51.06	51.80	52.32	51.81	50.81	1.10	0.62	0.24	0.03
a*	12.80^b^	12.87^b^	13.37^b^	14.77^ab^	16.04^a^	13.86^b^	0.71	0.02	0.33	0.03
b*	10.54	8.87	10.62	9.72	11.01	10.03	0.69	0.33	0.58	0.79
pH_45min_	6.96^a^	6.63^b^	6.61^b^	6.77^ab^	6.80^ab^	6.76^ab^	0.08	0.05	0.67	0.04
pH_24h_	6.23	6.22	6.22	6.28	6.30	6.34	0.05	0.42	0.07	0.44
Shear force (N)	16.46^a^	18.29^a^	12.35^b^	12.69^b^	12.22^b^	15.47^ab^	1.06	<0.01	0.01	<0.01
Drip loss%	11.20^a^	8.56^ab^	6.54^b^	7.53^b^	5.43^b^	6.51^b^	0.77	<0.01	<0.01	0.01
Leg muscle										
L*	48.12	50.00	48.42	47.48	47.77	47.62	0.98	0.50	0.54	0.92
a*	4.34	3.92	3.34	4.52	4.99	3.72	0.58	0.41	0.83	0.57
b*	11.17	9.78	8.31	8.90	9.25	8.65	0.65	0.05	0.06	0.10
pH_45min_	6.78	6.70	6.56	6.73	7.07	6.82	0.15	0.32	0.20	0.84
pH_24h_	6.62	6.60	6.68	6.67	6.60	6.52	0.06	0.59	0.22	0.18
Shear force (N)	12.91	13.83	12.26	12.91	12.26	16.08	1.06	0.14	0.21	0.08
Drip loss%	12.51^a^	8.17^ab^	5.31^b^	7.66^b^	5.13^b^	5.30^b^	1.06	<0.01	<0.01	0.02

Values are the means of one broiler selected from each replicate pen in one treatment (n = 6).

SEM, standard error of the mean; ANOVA, analysis of variance.

1) L*,a* and b* are indicators of meat color.

2) The experiment applied a corn and soybean-meal based diet which was supplemented with 0, 2%, 4%, 6%, 8% or 10% culture media of solid-state fermented *Isaria cicadae* in phase I (from d 1 to 21) for groups 1 to 6 respectively, and 0, 1.33%, 2.67%, 4.00%, 5.32%, or 6.67% in phase II (from d 22 to 42) for groups 1 to 6.

a,b In the same row, values with different letter superscripts mean significant difference (p<0.05), while with the same or no letter superscripts mean no significant difference (p>0.05).
